# Improving Shared Decision Making Between Patients and Clinicians: Design and Development of a Virtual Patient Simulation Tool

**DOI:** 10.2196/10088

**Published:** 2018-11-06

**Authors:** Simon Jacklin, Neal Maskrey, Stephen Chapman

**Affiliations:** 1 School of Pharmacy Keele University Keele United Kingdom

**Keywords:** clinical decision making, education, medical education, mobile phone, pharmacy education, virtual patient, virtual reality

## Abstract

**Background:**

Shared decision making (SDM) involves the formation of a collaborative partnership between the patient and clinician combining both of their expertise in order to benefit decision making. In order for clinicians to be able to carry out this skilled task, they require practice. Virtual reality, in the form of a virtual patient, could offer a potential method of facilitating this.

**Objective:**

The objective of this study was to create a virtual patient that simulated a primary care consultation, affording the opportunity to practice SDM. A second aim was to involve patients in the design of a virtual patient simulation and report the process of the design.

**Methods:**

We employed a multistep design process drawing on patient and expert involvement.

**Results:**

A virtual patient, following a narrative style, was built, which allows a user to practice and receive feedback; both clinical and communication skills are required for the simulation. The patient group provided multiple insights, which the academic team had overlooked. They pertained mostly to issues concerning the patient experience.

**Conclusions:**

It is possible to design a virtual patient that allows a learner to practice their ability to conduct SDM. Patient input into the design of virtual patient simulations can be a worthwhile activity.

## Introduction

Shared decision making (SDM) involves the formation of a collaborative partnership between a patient and clinician [1]. Clinicians know about clinical guidelines, basic science, their previous experiences, and case histories, while patients understand their experience of the disease, their lifestyle, what they prefer and expect as well as the risks they will tolerate [2]. Through communication, these 2 worlds can be combined to benefit the decision-making process [3]. This partnership is not necessarily equal at all times, that is, it does not have to be an exact 50%-50% contribution. Patients sit on a continuum, all holding disparate preferences for involvement in their care [4], but all these variants can be considered as shared if the dynamics between patients and clinicians are congruent. A patient may not want to make any final decision, but they should still be involved in the process, eliciting their concerns and views [5].

The push to encourage clinicians to practice SDM has ethical, legal, and clinical dimensions with respect to patients’ autonomy and their right to choose [6]. SDM with patients initiating treatment for inflammatory bowel disease was shown to increase patient satisfaction and likelihood of adherence to therapy and decrease costs [7]. Reduction in prescribing [8] and increase in patient satisfaction [9] and confidence in decisions made [10,11] have all been reported.

There are numerous barriers and difficulties inherent in influencing clinicians to utilize SDM more often and to the highest standard [12]. SDM is a skill and a potentially overlooked element is the fact that it requires training and development [13]. It could be easy for health care professionals to assume that by carrying out consultations in clinical practice, they are honing their abilities. This may not be the case as a key factor in the acquisition and development of skills is not just practice but feedback [14,15]. Routine clinical practice does not often allow the time for self-reflection or feedback from a senior or peer and so by itself is insufficient. This is compounded by the fact that clinicians themselves are not adept at identifying their own weaknesses [16].

So how can a clinician practice and receive this vital feedback? Current approaches have limitations; simulated patients are not standardized or accessible at all times; neither videotapes nor lectures and seminars are greatly interactive as the learner cannot make active choices to dictate the outcome of a case [17,18]. Ideally, what is required is a standardized, readily accessible, low-risk, and interactive method for practice and feedback. Advancements in technology mean that virtual reality can meet all of these criteria and offer a potential solution, specifically virtual patients (VPs).

VPs have been defined as a “specific type of computer program that simulates real-life clinical scenarios; learners emulate the roles of health care providers to obtain a history, conduct a physical examination, and make diagnostic and therapeutic decisions” [19]. They are standardized, safe, and tailorable; they permit repeated practice; and they offer new economies of scale.

The examples of VPs in the literature are a heterogeneous collection of technologies, perhaps due to different pedagogical aims. A significant differentiating factor is the type of skill they attempt to develop: technical or emotional. Technical skills include managing acute medical emergencies [20] and triaging patients [21]. More recently, there have been attempts to combine technical skills with emotional ones [22]; the cited example is somewhat different from others as it involves both patients and practitioners using the simulation. While the simulation touches on SDM, it focuses on other issues too, such as health education; the scenario is concerned with a patient requesting antibiotics when they are not required.

Many virtual cases reduce emotional skills to a technical exercise; the selection of a single question (eg, do you have any medical conditions?) liberates complete, sterilized answers from the patient. Conversation is not like this; there are interjections, misunderstandings, and clarifications. In addition to simplification of emotional skills, many VPs encourage the application of a treatment plan to a patient but not a discussion about the patient’s values and preferences to arrive at a decision that the patient and clinician are content with. Rote use of guidelines has previously been raised as a misapprehension of evidence-based medicine [23], and there are concerns that health care is becoming more data-driven, neglecting individual patient’s wishes [24]. Developments in virtual training for consultation skills need to address these concerns.

Patient and public involvement (PPI) is the activity of including patients and public in research as collaborators rather than as participants [25]. PPI is fast becoming a key feature of health care research [26]. Efforts have also been made to engage patients and laypeople in medical education as simulated patients, tutors, or advisors on curricula [27].

The aims of this study were to design and build a novel VP simulation for developing the dual skills of technical competence and interpersonal skills to make evidence-informed, shared decisions as well as to involve patient input in the design of the VP simulator.

## Methods

### Design

A multistep approach was taken with the design.

### Literature

The VP simulator was based on existing literature about what broad features make for a good consultation. The most common consultation model in the United Kingdom—Calgary-Cambridge [28,29]—was used as a loose structure for the script. Its 70 items provided the skeleton and flow for the simulation. The technical and clinical elements are based on National Institute for Health and Care Excellence (NICE) guidance.

### Initial Script Drafting

The initial script writing was completed by a pharmacist, medical doctor, and medicines optimization expert (SJ, NM, and SC, respectively). The script was branched, multiple-choice style; at each point, the user had 3 options to choose from to select what they wanted to say or do. There was then a corresponding patient response, and 3 more options were presented, and so on. The script was designed to allow the users to take circuitous routes through the consultation; for example, if early on, a key step is missed, the user could redirect the conversation back to pick up that key point. The VP is not based on a real patient. Any resemblance to persons living or dead is coincidental.

### Patient Involvement

Local patient advocacy groups were contacted to identify interested patients. Each of the 3 patients who agreed to participate was met individually and their initial ideas about the simulation discussed. Following initial script drafting, sections of the script were shown to each person and comments on realism and quality as well as any aspect of the script or simulation were elicited. Comments were collated, and the script was amended in the light of suggestions. A cycle of feedback from each patient was incorporated.

### Experts

The final phase in the development of the design, before the animation element, was an expert review. We asked 3 experienced primary care clinicians to interact with a prototype version of the tool and provide written feedback on their thoughts; the prototype was devoid of animation. Comments were invited on the clinical aspects as well as those relating to pedagogy, such as feedback. After the written reviews had been received, these were collated and the necessary amendments to the script were made.

### Technical Details

The script developed through these 4 stages was then built into a Web-based VP simulator; comments from patients and experts were focused on the script and nonanimated prototype. They did not review the animations and voice-over elements due to these being predetermined but did have an opportunity to review the image of the VP. Textbox 1 lists the particular specifications and products used to create the finished product.

**Development software used.**

- Script writing: Google Sheets and Docs, draw.io
- Audio editing: Adobe Audition and Adobe Premiere Pro
- Character and environment modeling, rigging, animation, and rendering: Maya 2014
- Character and environment texturing: Adobe Photoshop
- Compositing: Adobe After Effects CC
- Server-side scripting language: PHP

## Results

**Figure 1:**
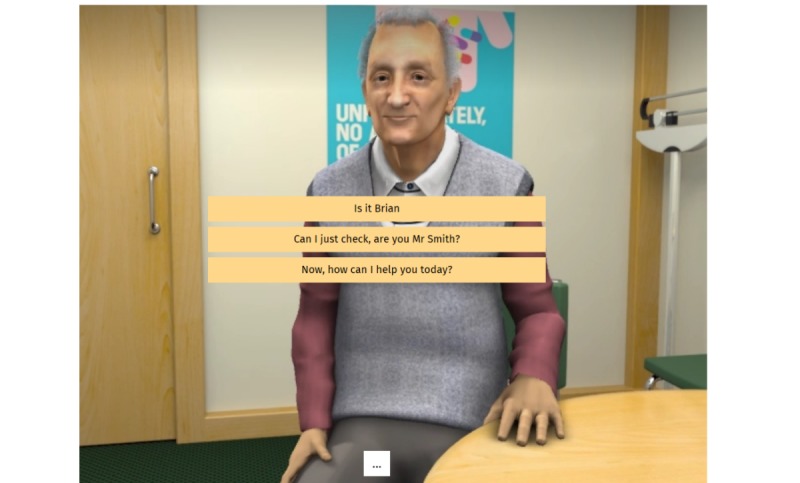
Screenshot of the virtual patient.

The setting was a primary care consultation room with the patient sitting in front of you (see Figure 1). Users play the role of either a general practitioner or a prescribing pharmacist, whichever is relevant to them. By selecting from the multiple-choice options, usually 3 each time, the user can navigate the scenario. After each option selection, the patient will respond with a reaction using both prerecorded speech and body language shown by high-quality animation. Sometimes the patient will not answer a question completely or will respond with a question of their own; this is to mimic a more natural style of communication. At the end of the simulation, the user will receive feedback. The simulation can be used on a computer, tablet, or smartphone.

The scenario is based on the decision as to whether to initiate a statin and a “hidden” patient agenda, such as an issue with the patient’s “waterworks.” The hidden agenda is to add realism to the simulation and to encourage the user of the simulator to conduct an open consultation and not one confined to their own agenda. If handled appropriately, the user should engage in two-way information exchange, discuss both risks and benefits, highlight the option to do nothing, and come to a decision based on the patient’s preferences. These are hallmark features of SDM [30]. The comments on the design from both the laypeople and expert clinicians are tabulated below in Tables 1 and 2.

**Table 1:** Patient feedback.

Laypeople comments	Resultant actions
Referring to the patient’s age is not relevant; polypharmacy is most likely the reason for the patient not wanting to take more medicines, which is irrespective of age.	Referring to age at this stage led to a negative feedback point.
An 84-year-old may well have hearing impairment so getting him to repeat back what changes he will make could be a way to check he has both heard and understood the discussion.	An extra chain of options was included that allowed a user to choose to do this.
There is no information on whether the clinician had met the patient before. Perhaps this information should be included at the start as it can affect the language used.	This information was included and feedback amended.
Patient background important as different cultures and ages affect communication.	Background was made more comprehensive, but this was balanced with reality; medical notes with full details of a patient’s social history were felt to be unrealistic.
In the sections where risk or benefit of treatment was discussed, it was felt that the softer approach with less numbers was good and should occur more within the script. Flexibility was also felt important as if the user delved straight into statistics; they should be able to “rescue” their attempt by providing a simpler follow-up explanation.	The possible route through the simulation was made more circuitous to allow users to make imperfect choices but then recover the situation later and vice versa.

**Table 2:** Expert feedback.

Expert comments	Resultant actions
No option to use a PDA^a^.	The option to use a PDA was included.
Very specific language used at certain stages, eg, different between “something versus anything.”	There is evidence to suggest subtle adjustments in language can have profound effects [31,32].
There could be the option of a middle ground when presenting risk; current options are too distinct.	A middle ground option was included so the choice of risk explanation language was not so dichotomous.
If the case is handled very poorly, there is little feedback.	More feedback was added in the event that a user handles the simulation very poorly.
Feedback at the end is given too quickly.	A pause was added between points and a written summary provided at the end.
Whether the statin is for primary or secondary prevention is not clear.	The patient’s medical history was amended to make it clearer.
The supposed red flag symptom is not clear enough.	A further bit of dialogue was added, making the urinary symptoms more explicitly a red flag.
Needs to be clear to the patient that we cannot predict whether they will or will not have a cardiac event.	A line was added to stress that we cannot predict in advance whether a person will experience an event.
No feedback for missing a potential red flag.	Additional feedback was added.
Wording of feedback could be more constructive.	Rather than stating a negative piece of feedback outright phrases such as “It was good you tried to...but...” were added to make them more constructive.
Medical notes not available from the start.	Amended such that the notes can be viewed at any given time.
Might be useful to have a print out of the feedback for use in development portfolios.	This function was added; a PDF of feedback can be downloaded each time the simulation is used.

^a^PDA: patient decision aid.

While not an explicit comment, many of the patients used the same words as those the VP used when replying to dialogue from the clinician; an encouraging sign that the language being used was lifelike.

In addition to the comments above (Table 2), additional suggestions were made about the technical elements of the simulation. These were not enacted but are listed as follows for future work: (1) feature a clock to show how long the consultation has been running, increasing the realism; (2) the ability to go backward in the consultation and retrace steps; and (3) have feedback given instantaneously, as the user goes along.

The feedback approach was based on the Kolb theory of reflection [33]; the theory is a common approach to simulation-based learning [34]. The simulation was designed to provide a concrete experience, which is the first step in the cycle. There was a short break between finishing the scenario and receiving feedback to allow some time for reflection. The feedback, given first by the VP and then in a text form, provided some points for reflection based on the user’s performance. To complete the cycle, the learning could be put into practice by repeating the simulation.

Due to this simulation trying to combine both technical and interpersonal competencies, as is the case in a real consultation, the feedback was broken down into two sections: emotional and technical. The patient animation gave the emotional, gentle feedback, for example, “you made me feel comfortable.” The technical feedback was provided in a written form, for example, feedback on initiating statin and specific wording choices. This results from some of the comments in Table 2. The feedback is exportable as a PDF to enable users to store a copy for use in portfolios.

To highlight the quality of the animation used, potentially important for the fidelity of the simulation, Figure 1 shows a screenshot of the case.

To experience the VP simulator, we have created a short YouTube video to provide a brief demonstration: “Virtual Patient Demonstration” [35].

## Discussion

### Principal Findings

By incorporating patient opinion and SDM principles, the resulting Web-based VP simulator simulated a clinical consultation congruent with a “real-life” situation. The entirety of the patient contact is simulated, from calling the patient into the room to the final remarks as they leave. Where a significant proportion of VP simulations have sought to develop a single skill or a set of skills, the user of this software must draw on the whole array of abilities required for a competent, patient-centered consultation.

A key difficulty in the initial phase of scripting, and indeed throughout, was the balance between the different multiple-choice options; one is good, one is bad and one is somewhere in-between. What constitutes wrong at a point in the consultation may be correct at another, and as stated, users can make a wrong decision but still bring the consultation back to a good conclusion. The difficult task was to make all 3 options plausible. This meant that all of the options were close enough together such that the choice was not obvious but not so close so as to be a “spot the difference” exercise. The patient involvement was very useful here.

Unpredictability emerged as a key theme during the patient involvement phase. It was a deliberate choice not to direct patients at the outset of their inclusion in the design process, allowing them to introduce elements that may not have occurred to the health care professionals in the design team. The authors, like all health educators and researchers, have a certain education, background, and set of experiences, which affect their perspective. Laypersons, though, have a different set of experiences, which means they can provide a different outlook or view on an issue. We cannot know what this perspective will be; hence, this is where the value of PPI or lay involvement is derived.

Early concern was that the patients involved would be overawed by the technology or the process and that they would not feel able to contribute anything. The opposite was also feared, a situation where the patient did not understand the aim of the design and continually suggested inappropriate modifications. What resulted was neither of these situations; all the patients clearly understood the aim and how they could assert their opinions and views.

### Conclusions

Involving patients in the design of VP simulations, particularly those involving any degree of communication, has been shown to be useful for creating realistic scenarios. The outputs from the involvement of patients cannot often be predicted, so it may well be a case of “try it and see.” While virtual reality simulations can be complicated and tricky to design, laypeople have the capacity to comprehend this and also contribute valuable ideas. We would recommend future VP designers to at least consider patient or laypeople involvement in their designs.

It is also possible to design a VP that encompasses both the technical and interpersonal elements of care. Many of the previous architects of these technologies seem to have stuck to one or the other, but to model reality more closely, both have been combined in this design. What has been created is a Web-based VP to allow repetitive practice and feedback for evidence-informed SDM. The next steps will be to evaluate and investigate the views of target users, namely under- and postgraduate health care professionals.

## Abbreviations

NICE: National Institute for Health and Care Excellence
PPI: patient and public involvement
SDM: shared decision making
VP: virtual patient
